# A Brief Review of the Shape Memory Phenomena in Polymers and Their Typical Sensor Applications

**DOI:** 10.3390/polym11061049

**Published:** 2019-06-15

**Authors:** Li Sun, Tao Xi Wang, Hong Mei Chen, Abhijit Vijay Salvekar, Balasundaram Selvan Naveen, Qinwei Xu, Yiwei Weng, Xinli Guo, Yahui Chen, Wei Min Huang

**Affiliations:** 1 School of Civil Engineering, Shenyang Jianzhu University, Shenyang 110168, China; sunli2000sy@163.com; 2 School of Mechanical and Aerospace Engineering, Nanyang Technological University, 50 Nanyang Avenue, Singapore 639798, Singapore; wa0003xi@e.ntu.edu.sg (T.X.W.); ABHIJITV001@e.ntu.edu.sg (A.V.S.); NAVEEN008@e.ntu.edu.sg (B.S.N.); QXU003@e.ntu.edu.sg (Q.X.); WENG0022@e.ntu.edu.sg (Y.W.); ChenYahui@ntu.edu.sg (Y.C.); 3 School of Materials Engineering, Jiangsu University of Technology, Changzhou 213001, China; 4 College of Chemistry and Materials Science, Sichuan Normal University, Chengdu 610066, China; chenhongmei@sicnu.edu.cn; 5 School of Materials Science and Engineering, Southeast University, Nanjing 211189, China; guo.xinli@seu.edu.cn

**Keywords:** shape memory material, shape memory polymer, sensor, anti-counterfeit, temperature sensor, wetting sensor

## Abstract

In this brief review, an introduction of the underlying mechanisms for the shape memory effect (SME) and various shape memory phenomena in polymers is presented first. After that, a summary of typical applications in sensors based on either heating or wetting activated shape recovery using largely commercial engineering polymers, which are programmed by means of in-plane pre-deformation (load applied in the length/width direction) or out-of-plane pre-deformation (load applied in the thickness direction), is presented. As demonstrated by a number of examples, many low-cost engineering polymers are well suited to, for instance, anti-counterfeit and over-heating/wetting monitoring applications via visual sensation and/or tactual sensation, and many existing technologies and products (e.g., holography, 3D printing, nano-imprinting, electro-spinning, lenticular lens, Fresnel lens, QR/bar code, Moiré pattern, FRID, structural coloring, etc.) can be integrated with the shape memory feature.

## 1. Introduction

Stimulus-responsive shape switching refers to the phenomenon that the shape change in a material is induced by an externally applied stimulus [1,2,3,4,5]. Such a kind of shape change might be reversible or non-reversible. The former is known as the shape change effect (SCE), and the latter is called the shape memory effect (SME) [6,7,8,9,10,11]. In fact, most polymers are heating- and chemo-responsive shape memory polymers (SMPs) as revealed in Reference [12], although the actual shape memory performance varies according to the actual polymer and its processing (programming and activation) method, and environmental conditions, etc. A polymer may have the SME or SCE according to, for instance, the relationship between its activation temperature and the environmental temperature where the polymer is used.

Let us take memory foam, which is a well-known name in the market and is mostly made of polyurethane (PU), as an example. At room temperature, memory foam cannot maintain its deformed shape that is technically defined as the temporary shape within the shape memory community. Memory foam slowly recovers its original shape, which is also called the permanent shape. It appears that at room temperature, memory foam has the SCE. However, if memory foam is deformed and then placed in the freezer part of a refrigerator, it is able to keep the temporary shape without any constraint applied, unless it is moved out of the freezer to be heated in the atmospheric air. Thus, memory foam does indeed have the thermo-responsive SME as well, but the working temperature for the heating-responsive SME is below the room temperature. The polymeric foam used by Crocs^TM^ essentially shares the same feature, i.e., its activation temperature (glass transition temperature, *T*_g_, for this particular polymer) is around room temperature. Hence, the heating-responsive SME in memory foam may be used as the mechanism for temperature sensors to indicate over-heating in cold chain logistics.

Although most of the research works on SMPs till now are about their potential actuator applications, this brief review is about their sensor applications, which is still a niche area and hence not so well explored [13,14,15]. While many new SMPs with a range of additional interesting features have been developed [16,17], since most polymers do have certain level of heating/chemo-responsive SME [12], herein we will mainly focus on applications utilizing commercially available polymers. Because these polymers are easily accessible at relatively lower cost and with higher quality, they are ready to be immediately applied into commercial products or integrated as part of commercial products.

In Section 2, a brief introduction of the working mechanisms for the SME in polymers is presented, together with the demonstration of various shape memory phenomena. This section provides the fundamentals for the applications of SMPs and some important issues, which need to be considered in actual applications, are also mentioned. In Section 3, we discuss the potential sensor applications based on in-plane and out-of-plane programming (pre-deformation), respectively. Section 4 is a summary, which includes the different approaches to utilize the SME in polymers for sensor applications, some typical commercially available SMPs and their suppliers, together with the major concerns in their real engineering applications and outlook for future developments. Main conclusions are presented in Section 5.

Unless citation is provided, the works presented here are based on our unpublished works.

## 2. Working Mechanisms and Shape Memory Phenomena

### 2.1. Working Mechanisms

**Figure 1:**
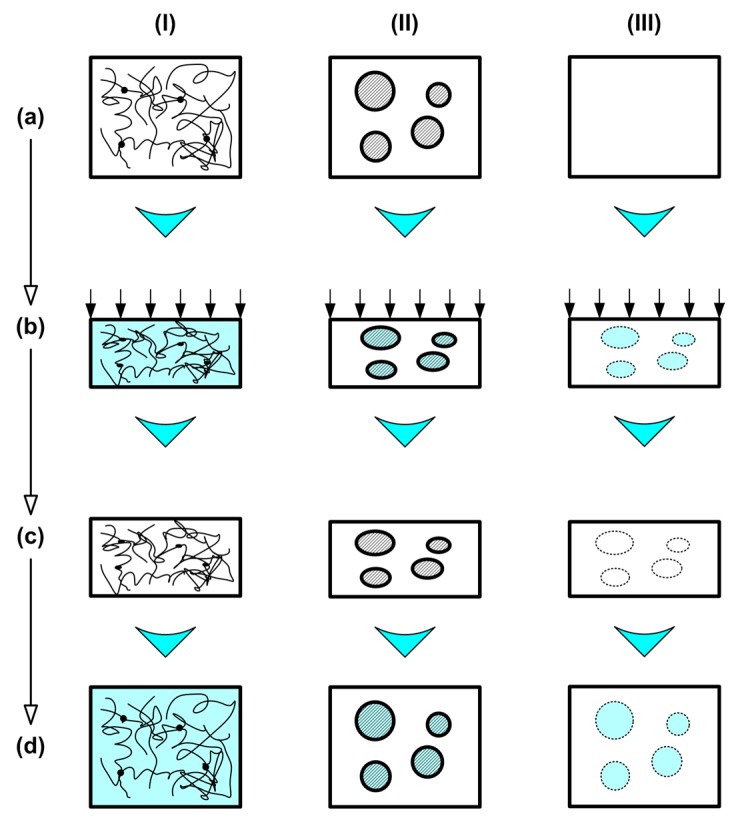
Basic working mechanisms for the heating-responsive SME in polymeric materials. (**I**) Dual-state mechanism (DSM); (**II**) dual-component mechanism (DCM); (**III**) partial-transition mechanism (PTM). (**a**) Original sample at room temperature; (**b**) upon heating and compressing; (**c**) after cooling and constraint removal; (**d**) after heating for shape recovery. (Modified from Reference [12]).

According to Reference [12], there are a few different working mechanisms for the SME in polymers. A brief discussion of the three basic ones for heating-responsive SMPs is provided here. Interested readers may refer to Reference [12] for a detailed explanation. Figure 1 illustrates the three basic mechanisms for the heating-responsive SME from programming for temporary shape fixing [(a)–(c)] to heating for shape recovery [(c)–(d)].

(I) Dual-state Mechanism (DSM)

In this mechanism, the polymer is heated to above its softening temperature (a), which is typically *T*_g_, so that it becomes soft and flexible, and thus easy to deform (b). After subsequent cooling for hardening, the constraint is removed, and the polymer is able to largely keep the deformed shape (c). This is called the programming process. Subsequently, the polymer is heated again for shape recovery (d). The steps from (c)–(d) are known as the recovery process, which is the second part of a full shape memory cycle after programming. 

(II) Dual-component Mechanism (DCM)

For an inclusion-matrix system, as illustrated in (a), if the matrix is always elastic, while the inclusions become softer upon heating, the material can be deformed easily at high temperatures (b). After cooling to harden the inclusions, the free-standing material is able to keep the deformed shape (c), unless it is heated again for shape recovery (d). Semi-crystalline polymer, in which the crystalline part could be considered as the inclusion, is a typical example. Polymeric shape memory hybrid (SMH), which is composed of at least two parts, but neither of them has the SME as an individual, is also under this mechanism.

(III) Partial-transition Mechanism (PTM)

If a polymer is uniformly heated for an incomplete transition, which softens it partially, the unsoftened part of the polymer may be considered as the matrix, which can be elastically deformed within a certain range [(a)–(b)]. After cooling to harden the polymer, the deformation is able to be largely kept (c), unless it is uniformly reheated again for shape recovery (d).

Above mentioned are the three typical working mechanisms for the heating-responsive SME in polymers. In any mechanism for the stimulus-responsive SME in polymers, there are normally two basic parts within the material. One part (elastic part) is always highly elastic, which stores elastic energy after programming, and the stored elastic energy provides the required driving force for subsequent shape recovery. The other part (transition part) is able to change its stiffness, depending on whether the right stimulus is applied, and after programming, the deformation is plastic or quasi-plastic. Some ductile polymers can be programmed without heating to above their softening temperature, since their transition part is able to significantly deform in a plastic or quasi-plastic manner at low temperatures without early fracture [18,19,20].

The evaluation of the shape memory performance in polymers includes two important parameters, namely, the shape fixity ratio (*R*_f_, after programming) and shape recovery ratio (*R*_r_, after recovery). However, due to the influence of visco-elasticity, which varies according to the exact polymer, we may need to consider both the short-term (instant) and long-term response under the testing environment and programming conditions [21]. Herein, we take programming via uniaxial stretching at high temperatures as an example. A piece of SMP is heated for softening and then stretched to the required programming strain (*ε*_pro_). Subsequently, the sample is cooled to room temperature with the programming strain of *ε*_pro_ fixed. Immediately after the removal of the constraint, a residual strain of $\varepsilon_{\mathrm{res}}^{1}$ is obtained. Since some polymers may creep/relax significantly before a particular stimulus is applied for shape recovery, long-term residual strain ($\varepsilon_{\mathrm{res}}^{2}$) should be used for them. This ends the programming process, and the short-term shape fixity ratio ($R_{f}^{S}$) and long-term shape fixity ratio ($R_{f}^{L}$) are defined by,

(1a) $$R_{f}^{S}=\frac{\varepsilon_{\mathrm{res}}^{1}}{\varepsilon_{\mathrm{pro}}}$$

and

(1b) $$R_{f}^{L}=\frac{\varepsilon_{\mathrm{res}}^{2}}{\varepsilon_{\mathrm{pro}}}$$

respectively. In the next recovery process, the programmed SMP is heated, and the remaining strain after heating is defined as *ε*_rem_. Thus, the shape recovery ratio (*R*_r_) is expressed as,

(2) $$R_{r}=1-\frac{\varepsilon_{\mathrm{rem}}}{\varepsilon_{\mathrm{res}}^{i}}$$

where *i* is 1 or 2. 

While high shape recovery strain, high shape fixity ratio and high shape recovery ratio are ideal in many applications, they are not always required. For instance, in surface wrinkling via deposition of an elastic thin layer atop a piece of one-directional pre-stretched polymeric substrate, the only requirement is a few percent of strain recovery [22]. A high recovery strain indeed results in cracking of the elastic layer in the transverse direction due to over stretching during shape recovery in the SMP substrate, and both the shape fixity ratio and shape recovery ratio are not necessary to be very high. In fitting applications of wearable items based on elastic SMPs [23], 100% shape fixity ratio is not good as the corresponding gripping force approaches zero. A small amount of elastic stress due to incomplete shape recovery is ideal. The required exact amount of elastic stress depends on the actual application.

### 2.2. Shape Memory Phenomena

The SME, in particular the heating-responsive SME, of many commercial polymers has been characterized (e.g., in [24,25,26,27,28,29,30] for polycarbonate (PC), poly(ether ether ketone) (PEEK), ethylene-vinyl acetate (EVA), polytetrafluoroethylene (PTFE), poly-ε-caprolactone (PCL) and poly(methyl methacrylate) (PMMA)). 

Here, we take PMMA (also known as acrylic, which is a widely used engineering polymer, including in optical engineering) as an example to explain various shape memory phenomena in polymeric materials. In addition to actuator applications, those phenomena, together with the different approaches for programming to fix the temporary shape, could be utilized in sensor applications as well.

**Figure 2:**
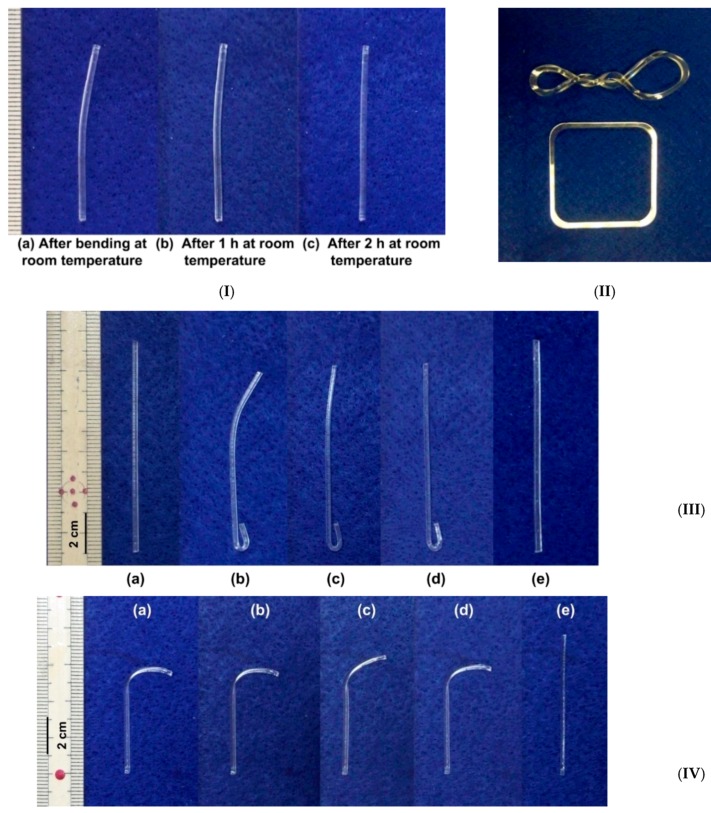
Shape recovery in poly(methyl methacrylate) (PMMA) via different ways of pre-deformation. (**I**) Relaxation in slightly bent (at room temperature) sample. (**II**) Heating-responsive shape memory effect (SME) in sample programmed at high temperatures (above *T*_g_). Top: after programming; bottom: after heating. (**III**) Comparison of the influence of programming conditions on recovery upon heating. (**a**) Original shape; (**b**) after bending (bottom part: bent above *T*_g_, top part: bent in 95 °C water); (**c**) after heating in 90 °C water; (**d**) after heating in 98 °C water; (**e**) upon further heating to over *T*_g_. (**IV**) Triple-SME. (**a**) After heating to above *T*_g_ and bending down; (**b**) heating in 98 °C water; (**c**) bending upon slightly in 98 °C water; (**d**) heating in 98 °C water; (**e**) heating to above *T*_g_. ((**a**–**c**) is the programming process, (**d**–**e**) is the recovery process.) [(**III**) and (**IV**) are reproduced from Reference [30] with permission].

In Figure 2, the shape recovery of a commercial PMMA is investigated after being pre-deformed (programmed) in different ways. In Figure 2I, the sample is slightly bent at its top end at room temperature, which is about 22 °C. It is able to gradually return its original straight shape, after being left at room temperature for two hours. This is indeed the SCE, as the programmed shape is not stable at room temperature. In Figure 2II, the sample is heated to over its *T*_g_, which is about 110 °C, and twisted (top piece). Recovery back to the original shape (bottom) only happens when it is heated again. In Figure 2III, the two ends of a piece of PMMA are programmed via bending under different conditions. The top end is bent in 95 °C water, which is below its *T*_g_, while the bottom end is bent at above *T*_g_. The top end is bent much less than the bottom end (but more than that in Figure 2I, as the material is still brittle at 95 °C (a–b)). Upon immersing the whole piece of sample into 90 °C water and then 98 °C water, the top end bends back to the original straight shape in a step-by-step manner, while the bottom end shows almost no shape change (c–d). Upon further heating to over *T*_g_, the bottom end becomes straight (e). In Figure 2II,III, shape recovery is triggered by heating and is always from a programmed shape, which is temporary, to the original shape, which is permanent. This is also called the dual-SME, since there are only two stable shapes in the shape memory cycle. In Figure 2IV, the PMMA sample is programmed in two steps. Its top end is bent to the right (down) above *T*_g_ (a), so that upon heating in 98 °C water, there is not any apparent shape change at all (b). In the next programming step, the top end of the sample is bent slightly to the left (up) in 98 °C water (c). The following recovery process also includes two steps. Upon heating in 98 °C water, the top end bends to the right (down) (d). Upon further heating to over *T*_g_, the sample becomes straight (e). We can clearly observe a stable intermediate shape between the temporary shape and the permanent shape in this case. This is called the triple-SME. The triple-SME can be achieved in polymers with two transitions (e.g., the glass transition and melting/crystallization) or one transition (glass transition or melting/crystallization) via two-step or one-step programming [18,19,27,31,32,33,34,35,36,37,38,39,40,41]. It is possible to have more than one intermediate shape via multiple-step programming to achieve the multiple-SME.

As discussed in Reference [12], the two important programming parameters, namely programming temperature and maximum programming strain, have significant influence on both shape fixity ratio and shape recovery ratio, which are two key parameters in the characterization of the shape memory behavior of a SMP.

**Figure 3:**
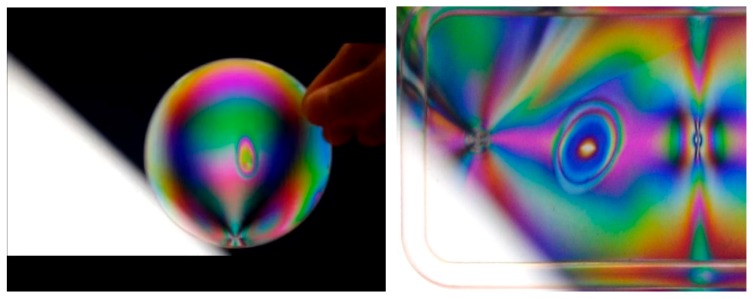
Heating to alter the color pattern observed via photo-elasticity. (**Left**) plastic petri dish; (**right**) PP box (cover). The circular/elliptical areas are produced by local heating to alter the internal stress induced by injection molding.

Vitrimer is a term for those polymers, in which cross-linking can be opened/closed under certain conditions (mostly via heating/cooling) in a reversible manner [42,43]. Consequently, a vitrimer polymer is thermo-set at low temperatures, and becomes thermo-plastic at high temperatures. Recently, a vitrimer-like SMP has been used for so called digital coding of mechanical stress [44], in which the elastic stress field introduced by programming can be altered. Therefore, the color pattern observed via photo-elasticity can be tailored. Such a technology may be applied in anti-counterfeit applications. This kind of color pattern change can be realized in conventional polymers as well via local heating. Figure 3(left) is a piece of plastic petri dish, and Figure 3(right) is polypropylene (PP) box (cover). Both are produced by the same process of injection molding, but we can see different color patterns via photo-elasticity. The circular shaped areas in the middle of the two plastic pieces in Figure 3 are produced by local heating using a lighter. The advantage of using vitrimer is to minimize, if not totally avoid, the flow induced shape change during heating of the conventional thermo-plastic polymers to their melting temperature. The special feature of vitrimer, i.e., the reversible cross-linking, ensures that all vitrimers have good heating-responsive SME when they are in the cross-linked state. Many actuator and sensor applications using vitrimer are expected. As mentioned above, since the focus of this review is mostly on commercial polymers, we will not explore the topic of vitrimer further.

### 2.3. Further Discussions

In this subsection, a couple of important issues in applying SMPs in engineering applications are briefly addressed.

Here, polymers used in 3D printing are taken as examples, as 3D printing is expected to reshape manufacturing in many ways, from individual fabrication (customized manufacturing) to fabrication of those items, that the traditional technologies have some difficulties to fabricate (e.g., those products that require inner mold). 3D printing is able to provide unique shape/surface feature for each individual piece produced in a cost-effective manner [45], so that 3D printing can be used in, for instance, various anti-counterfeit applications, as well. Since printed electronic circuits are actually a 2D version of 3D printing [46], a combination of printed circuit and SMP is a natural extension.

At present, fuse deposition modeling (FDM) is able to provide a cost-effective solution for 3D printing. The most popular filaments for 3D printing via FDM at present include poly(lactic acid) (PLA), which is biodegradable, and acrylonitrile butadiene styrene (ABS), which is a well-recognized high-quality engineering polymer. Both materials have been confirmed to have excellent heating-responsive SME [47]. SMP Technologies, Inc., Japan has recently brought its shape memory PU filament into the market for 3D printing based on its well-known MM series of thermo-plastic shape memory PU [48]. In many aspects, polyetherimide (PEI) is similar to PEEK, which is a widely used engineering polymer, in particular, for high temperature applications, while PEI is relatively cheaper than PEEK. Both PEI and PEEK filaments for 3D printing are commercially available in the market, but a special high temperature 3D printer is required for printing both of them, as the maximum heating temperature for most of the conventional FDM printers is below 250 °C, which is below the printing temperature required for PEI and PEEK. Excellent heating-responsive SME at high temperatures is expected in the printed items using these high temperature polymers. On the other hand, PCL filament must be printed at 100 °C or below, as heating to high temperatures causes permanent damage to PCL, while programming of 3D printed PCL items via FDM must be done at low temperatures (below its melting temperature) [29].

Polyvinyl alcohol (PVA) is water soluble and thus, it is widely used in 3D printing as the material to print support structures, which can be removed later by water. It has good heating-responsive SME and its water-responsive SME is also demonstrated, if wetting is minimal before dissolving occurs [47].

Although a few other engineering polymers have the water/moisture-responsive SME [49,50,51] and do not either dissolve or significantly swell in water, they are mostly unable to respond quickly, unless they are in the shape of a very thin film or a very thin-walled foam.

While hydrogel is featured by its ability to absorb a large amount of water, that may cause significant volume expansion, i.e., swelling, dry hydrogel essentially belongs to the polymer category. Therefore, we can use either water or moisture as plasticizer to reduce *T*_g_ and thus trigger shape recovery [52], i.e., chemo-responsive SME, in dry hydrogels. This feature can be utilized for water/moisture wetting sensors [53]. It should be mentioned that depending on the exact water content, a piece of hydrogel may have the SME or SCE. Interested readers may refer to Reference [53] for a detailed explanation. Furthermore, since water penetration is a gradual process, the reaction time of a piece of programmed dry hydrogel is not only material dependent, but also sample size dependent. Thus, we may utilize this feature to achieve time-controlled reaction. As reported in Reference [54], upon immersion in water, the buckling starting time of a piece of pre-stretched cross-linked poly (ethylene glycol) (PEG) filament can be tailored by varying the diameter of the dry hydrogel filament and the amount of pre-strain. 

Similar to a lot of conventional polymers, many 3D printing polymeric materials (not only limited to those thermo-plastic polymers used in FDM, but also those cross-linked via, for instance, light curing) appear to be easier to relax/creep due to the well-known reasons, such as, softening upon absorption of moisture (plasticization), and molecular chain reorganization (micro Brownian motion) [55]. Since the heating-responsive SME in most of the 3D printed polymeric items is based on *T*_g_, and the choice of polymers for printing is still limited at present, it appears to be a very important concern in material selection for 4D printing, in which the additional D refers to the capability of shape change in 3D printed items, and of course, this feature could be used in anti-counterfeit applications as well. 

In the early stage of materials selection for sensor applications of SMPs, we may reveal the stability of a particular polymer via differential scanning calorimetry (DSC) test without introducing any macroscopic shape change [56,57]. The equivalent time-temperature effect (the time-temperature superposition principle), which considers the accumulation of both heating/cooling temperature and heating/cooling time, may be used in temperature-time accumulation sensors after careful and extensive calibration. But this part is not within the scope of this review. 

**Figure 4:**
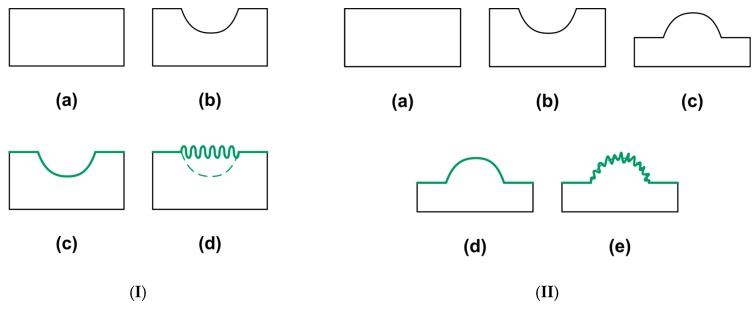
Two typical concepts of shape memory composites with elastic coating atop. (**I**) Indentation-coating-heating. (**a**) Original sample; (**b**) after indentation; (**c**) after surface coating; (**d**) after heating for shape recovery. (**II**) Indentation-polishing-heating-coating-heating. (**a**) Original sample; (**b**) after indentation; (**c**) after surface polishing; (**d**) after coating; (**e**) after heating.

According to Reference [1], a shape memory composite should have at least one component with the SME. Two typical concepts of elastic coating atop a polymer are illustrated in Figure 4. In Figure 4I, the surface of a piece of polymer (Figure 4I(a)) is impressed to form an indent (Figure 4I(b)). Subsequently, a layer of thin elastic film (e.g., Au) is coated atop (Figure 4I(c)). After heating for shape recovery, wrinkles are formed within the indented area, which at a macroscopic scale, is now a flat surface due to the SME (Figure 4I(d)). Structural coloring effect [58,59] may be observed, if the wavelength and magnitude of the wrinkles are optimized [13,60]. However, as mentioned above, if the coating is brittle and the recovery strain of the underneath polymer is too big, cracks may be resulted in the direction perpendicular to the wrinkles [61]. An effective way to get rid of this cracking problem is to have the same polymer coated atop [62,63]. In Figure 4II, after impression to form an indent atop a piece of polymer, its surface is polished to fully or partially remove the indent. Thus, after heating, a protrusion is formed (Figure 4II(c)) [64]. After coating (Figure 4II(d)) and then heating, wrinkles are formed on the surface of the protrusion (Figure 4II(e)) [65]. The formation of such wrinkles requires higher heating temperature and the exact temperature required for wrinkling is a function of the radius of the protrusion [61], which might be utilized to monitor over-heating temperature.

Currently, there are two types of temperature memory effect (TME) in SMMs (particularly, for shape memory alloys and polymers). One is without any macroscopic shape change, and the other is based on the shape memory phenomenon. The former mostly associates with some specific features revealed by DSC with the highest heating (or lowest cooling) temperature in the previous heating (or cooling) process [66]. The latter is mostly applicable to polymers and is meant to find the relationship between the remarkable or even full shape recovery (upon heating) and the previous programming temperature [14,18,19,39,40,41,67,68,69,70]. As revealed in Reference [12], both maximum programming strain and programming temperature are important parameters to determine the actual level of shape recovery upon heating. In the case of small maximum programming strain, most significant recovery or even full recovery occurs upon heating to the previous programming temperature, provided it is between the transition start temperature (*T*_s_) and the transition finish temperature (*T*_f_), or slightly above it. On the other hand, if the maximum programming strain is large, shape recovery shifts to higher temperatures. 

As observed in shape memory alloys, thermally induced reversible SME has also been achieved in some semi-crystalline polymers [71,72,73,74,75,76,77,78]. According to the above definition, strictly speaking, such a reversible SME upon thermal cycling should be under the category of thermo-responsive SCE. This review will not cover this topic.

## 3. Typical Sensor Applications

The SME has added one more dimension to potentially widen the application field of SMPs, since we can utilize this feature to design a smart polymeric system to react automatically to the change of the surrounding environment in a passive manner [79,80]. However, as mentioned in Section 1, so far, the number of successfully developed SMP products is still rather limited in the commercial market. A couple of commercial products relevant to sensor applications are listed here. The materials used in these applications do have the potential to be used in sensor applications as well, if a right application is identified.

**Figure 5:**
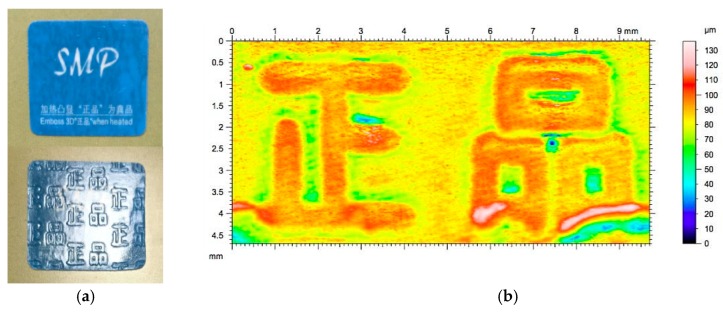
Typical anti-counterfeit shape memory polymers (SMP) label (0.16 mm thick) available in the market (from Guangzhou Manborui Material Technology Co., Ltd., Guangzhou, China). (**a**) Top: before heating (as received); bottom: after heating in an oven at 65 °C for two minutes. (**b**) 3D surface scanning result (using Taylor scanner), which reveals that the surface feature after heating for shape recovery is about 100 μm in height.

Figure 5a presents a commercial anti-counterfeit SMP label (from Guangzhou Manborui Material Technology Co., Ltd., Guangzhou, China) before and after heating in 65 °C oven for two minutes to get the embossed feature recovered. 3D surface scanning using a Taylor scanner reveals that the embossed feature, which indicates authentic in Chinese, is about 0.1 mm in height, which is about the same magnitude as that of the surface feature in standard bank coins and can be clearly identified via tactile sensation by fingers.

Shape memory polymeric splints are mostly made of poly-ε-caprolactone (PCL) type of polymers with/without cross-linking [29]. After heating for softening, they can be programmed at body temperature for fitting. After fitting, they become hard and stiff to provide strong support. 

**Figure 6:**
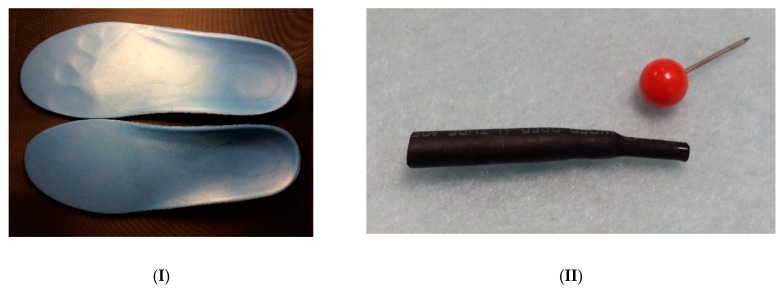

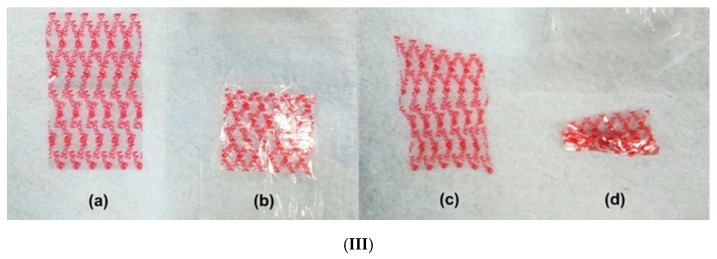
(**I**) Shape memory ethylene-vinyl alcohol (EVA) insole. Top: after heating and stepping on, foot shape is captured after cooling back to room temperature; bottom: after heating for shape recovery. (**II**) EVA heat shrink tube. The right end is heated for shape recovery. (**III**) Commercial heat shrink label. (**a**) Original; (**b**) after heating (placed between printing transparency and tape during heating); (**c**) after heating (placed between two steel plates fixed by two clips); (**d**) after heating (free standing).

Contrary to the memory foam, soft and elastic SMP foam is able to simultaneously achieve comfort fitting and provide enough support without compromising in elasticity [23,81]. Hence, a piece of insole made of such a type of SMP foam (mostly using either ethylene-vinyl acetate (EVA) or PU) is able to maintain the shape of the foot after stepping on it for a while during cooling for personalization (Figure 6I, top piece). The foot impression can be fully removed after heating for reus (Figure 6I, bottom piece).

Probably the top two most successful commercial SMP products at present are heat shrink tubes for protection of cables/wires and heat shrink films (membranes) for label and packaging. Although different types of polymers are used, mostly they are cross-linked via irradiation. In Figure 6II, the right end of a piece of heat shrink tube, which is made of cross-linked EVA, is heated to over 80 °C, which is above its *T*_g_ [26], so that this part shrinks remarkably. Figure 6III(a) is a piece of commercial heat shrink label (with screen-printed protruding oil on one side of its surface), and Figure 6III(b–d) present the shapes of the labels after heating under three different restrained conditions, from free-standing to restrained between two rigid plates clipped together. After heating for shrinkage of the label, strip-wrinkles are formed in the area coated with protruding oil due to buckling. Consequently, if the label is heated for free recovery [i.e., without any restraint as in Figure 6III(d)], instability in the form of a combination of local buckling (wrinkling) and global buckling is resulted.

According to the previous definition, the SME is meant for non-reversible, one-time activation. Although the SME cycle can be repeated after re-programming, if the programming process is practically not easy without causing any damage or misalignment, etc., sensors based on SMPs have the combined advantages of having high reliability/security and being non-repeatability/non-reusable. 

An extremely important issue in any real engineering application is the cost-effectiveness of a product. The cost (including both material cost and processing cost) of some above-mentioned successfully commercial products, such as heat shrink tube, EVA/PU foam, and PCL splint, has been widely accepted by the market, since the functions that those products provide are well worth their values. However, for a new application field, for instance in anti-counterfeit labels, the competitors are other materials and technologies, which are mostly well-developed/established and low price, new unique functions must be identified to justify the high price in using SMP based alternatives.

Herein, we will limit our discussion to the SMP-based sensor applications without the consideration of the equivalent time-temperature effect (although there are commercial sensors, for instance, Timestrip from Timestrip UK, Ltd., for this kind of purpose), and focus mostly on using commercially available polymers. Those special polymers with gradient properties, such as gradient transition temperature [16] and above-mentioned vitrimer, are excluded. Mullins effect and Payne effect, etc. in rubber-like elastic polymers are also ignored [82,83,84,85]. From application point of view, film and thin sheet should be more convenient in many sensor applications, such as SMP based anti-counterfeit labels reported in References [86,87,88] and Figure 5. We will focus on heating/wetting sensors and anti-counterfeit labels based on heating and water-responsive SME. 

A piece of SMP film/sheet may be programmed in the in-plane direction (load applied in the length/width direction) or out-of-plane direction (load applied in the thickness direction). Readers may refer to, for instance, References [25,53,89], for the details of various approaches to program different types of polymeric materials.

In order to provide the same feature height of 0.1 mm as in most of coins (refer to Figure 5), so that we can feel by fingers, the thickness of Manborui’s anti-counterfeit SMP label is 0.16 mm, which is far thicker than most commercial anti-counterfeit labels in the market. The typical thickness of hologram label is 40 μm, while the typical thickness of void paper is 100 μm. In-plane programming may be done for any thickness of film/sheet. However, to reduce the material cost, thinner film is preferred in most of the cases. On the other hand, for out-of-plane programming, we need to consider both the cost of material and processing (mostly programming via impression). In the case of utilizing the structural coloring effect via nano-imprinting, although we can minimize the thickness of SMP to reduce the material cost, the mold is actually the most expensive part. An ordinary mold produced by CNC machining or even 3D printing is much cheaper and well-suitable for tactual sensation by finger touching. Thus, the SMP film must have enough thickness in order to be stiff enough to maintain the surface feature upon finger touching.

### 3.1. In-Plane Programming

In-plane programming may be done in one or two directions during processing in, for instance, roll-to-roll fabrication of films/membranes.

Heat-shrink films and tubes are commercially available with different activation temperatures and mechanical properties. Typical polymers used are polyethylene (PE), multi-layer co-extruded polyolefin shrink film (POF), oriented polystyrene (OPS), PET, polyvinyl chloride (PVC) for heat shrink films and radiation cross-linked EVA for heat shrink tubes. Biaxially-oriented PET (BOPET) is the typical substrate material for hologram stickers. The technique to fabricate heat shrink films/tubes with zero Poisson’s ratio upon heating for shape recovery has been widely applied. 

**Figure 7:**
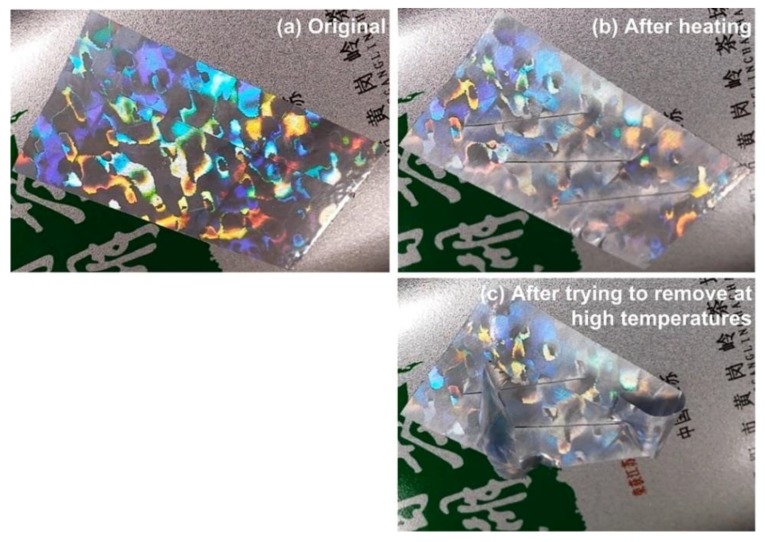
Cracking in hologram sticker upon heating. (**a**) Original label, in which no apparent crack can be observed and the color appears vivid; (**b**) after heating by hair dryer, cracks are apparent and the color turns dull; (**c**) removal at high temperatures results in tearing of the label.

To prevent tampering, for instance, heating to remove a piece of hologram label or void paper without causing any damage to the label/paper, the anti-heat transfer function should be integrated into the original label/paper to have, for example, self-destroy function upon heating. Since the heating-responsive SME is an intrinsic property of most polymers [12], we should be able to easily integrate this effect into currently used polymers in anti-counterfeit applications to enhance the reliability of security. In Figure 7, a very sharp blade is used to cut lines atop a piece of commercial hologram sticker. These lines are not easily visible by naked eyes (Figure 7a). After heating using a hair dryer, these lines open wider, so that we can easily see and thus the label is damaged and the original vivid color turns dull, most likely due to heating induced surface recovery (Figure 7b). These pre-cut lines serve as a way to prevent removal of the label at both low and high temperatures, which is able to effectively prevent heating and then the subsequent removal of the anti-counterfeit labels (Figure 7c). This approach can also be integrated into conventional void paper to prevent tampering at high temperatures. 

**Figure 8:**
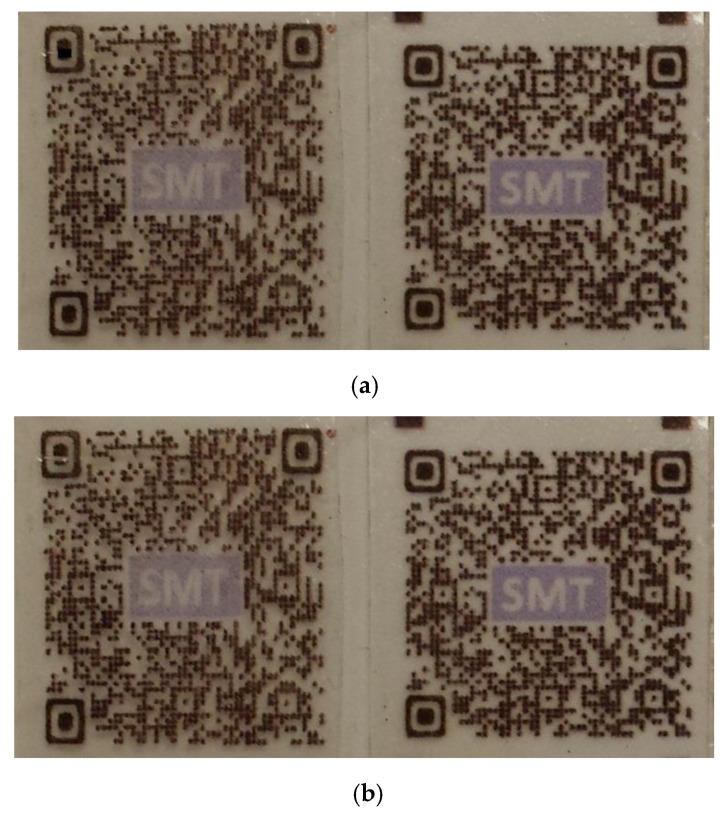
Anti-heat transfer quick response (QR) code. Left: modified label; right: without modification which is always readable. (**a**) Before heating, both are quickly readable by smartphone; (**b**) after heating, the left is unreadable or takes much longer time to read, while the right is still quickly readable as before.

Due to the fast readability and greater storage capacity, quick response (QR) code has become a very popular way right now in product tracking, item identification, time tracking, and document management, etc. The same heating-then-cracking concept shown in Figure 7 can be applied to result in anti-heat transfer QR code for improved security as well. As demonstrated in Figure 8a, before heating, both QR labels with/without modification can be easily identified by a smartphone with QR code scanning function. After heating, a small line crack appears on the upper left corner of the modified label (Figure 8b). Depending on the exact software used to read the QR code, some take longer time to read the code, while some just cannot recognize the code anymore. Of course, with further modification (e.g., with more pre-cut cracks and larger programming strain), we can make the QR code completely un-readable after heating.

**Figure 9:**
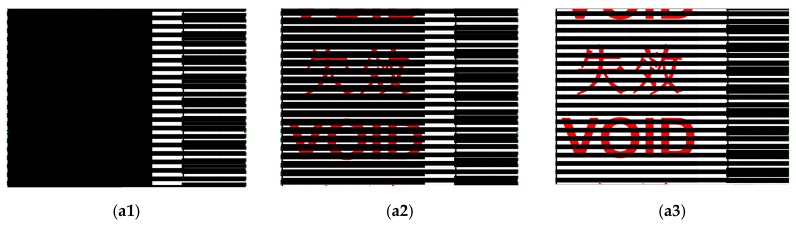

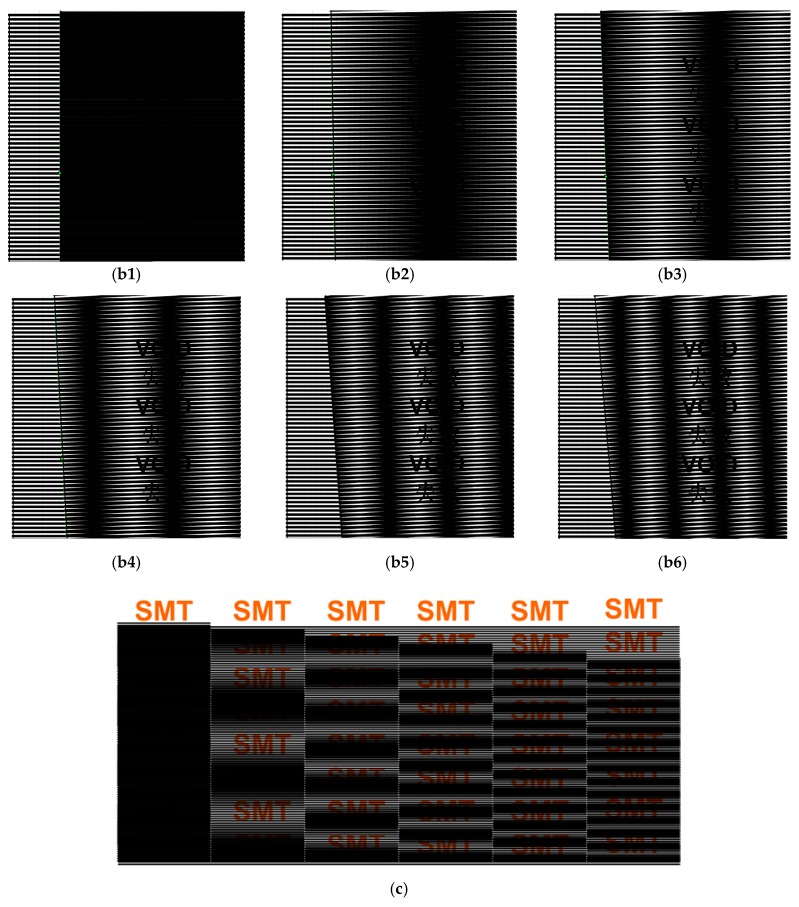
Moving Moiré pattern based on the shape memory effect (SME) upon gradual heating. (**a**) Vertical shifting of the top layer upon gradual heating; (**b**) rotating of the top layer upon gradual heating; (**c**) vertical shrinking of the top layer upon gradual heating.

Stripe interference between two layers with patterns printed atop (Moiré pattern) is another possible application using SMP to produce the moving effect during heating induced shape recovery. Three examples are illustrated in Figure 9. In Figure 9a, upon step-by-step heating, the top layer, which is transparent, moves up gradually, while in Figure 9b, upon heating, the top transparent layer rotates slightly. Figure 9c is different from above two cases, as the top layer shrinks upon heating. A closer look reveals that there is a fixed relationship between the number of black bands and the actual strain due to shrinkage. Hence, after calibration, this phenomenon may be used in strip shaped temperature sensors to monitor the maximum heating temperature.

**Figure 10:**
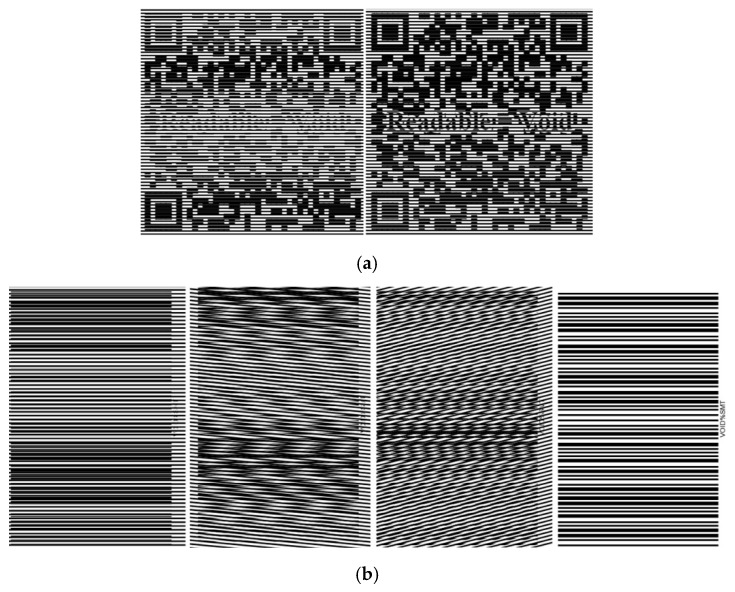
Anti-heat transfer quick response (QR) code (**a**) and barcode (**b**). (**a**) Left: before heating (unreadable); right: after heating (readable to show void). (**b**) Left three: before heating (unreadable); right: after heating (readable to show void).

Figure 10 demonstrates two possible ways to integrate the heat shrink function with QR code and barcode, respectively, to add in anti-heat transfer function into the original codes. As shown, after heating, both codes become readable and indicate void when scanned to show that both labels have been tampered by heating.

**Figure 11:**
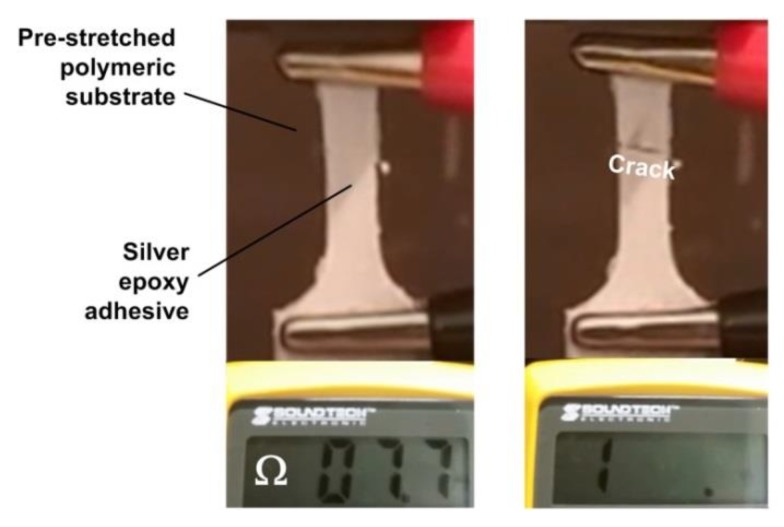
Break of circuit if over-heated. (**left**) Before heating, the measured electrical resistance is low (0.77 Ω); (**right**) after heating, the resistance is infinite.

Above mentioned heating-then-cracking function has been integrated into an electrical circuit. Thus, if over-heated, the circuit breaks, as demonstrated in Figure 11, in which silver epoxy adhesive is coated atop a piece of pre-stretched polymeric substrate with a narrow crack produced by a sharp blade.

**Figure 12:**
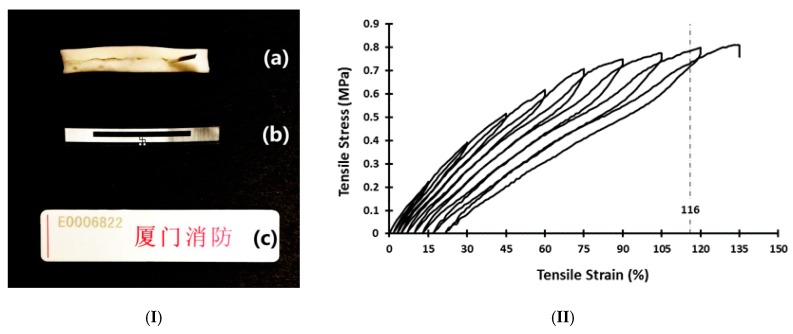
Stretchable tag with embedded radio-frequency identification (RFID). (**I**) Prototype of stretchable tag with embedded RFID (**a**) (sliced from middle to reveal the embedded RFID), free-standing RFID itself (**b**) and current flexible tag with embedded RFID (same RFID as in b). (**II**) Tensile stress vs. tensile strain relationship of stretchable tag upon cyclic stretching. 116% is the strain that the embedded circuit breaks.

Radio-frequency identification (RFID) is another very popular communication method at this moment, which uses electromagnetic fields to automatically identify and track tags attached to objects. Near field (NF) communication, which has become one of the built-in functions in most of current smartphones, is a special type of RFID. A silicone tag (Figure 12I(c)) is embedded with RFID (Figure 12I(b)) in the middle. Since silicone is elastic and the piece of RFID is placed in the middle of the tag (neutral plane), the tag is flexible for bending, but it is designed to be neither stretchable nor twistable, as both types of deformation trend to break the embedded RFID. By replacing elastic silicone with highly elastic SMP [84,85] and inducing wrinkles [65] in the piece of embedded RFID, the tag in Figure 12I(a) becomes stretchable. In Figure 12II, the stress vs. strain relationship in cyclic stretching of a piece of stretchable tag is presented. The measured electrical resistance in the embedded electrical circuit remains constant, until it is stretched to 116%, which is slightly less than the designed circuit failure strain of 120%. The major advantages of utilizing elastic SMP over the elastic silicone are twofold. One is easier to stretch more at high temperatures in SMPs, and the other is without the need to keep stretching during fabrication. One more additional advantage is the repeatable comfort fitting of, for instance, wearable electronic devices, to match the shape of each individuals at the spot to realize one-size-for-all at low cost [23,81].

### 3.2. Out-of-Plane Programming

The SME in polymeric materials is able to provide not only visible (to see), but also tactile (to feel) change after shape recovery. The latter function is lack in most of the current anti-counterfeit labels, except the shape memory label in Figure 5, in which a special polymer with a *T*_g_ around 60 °C is used. The unique tactual sensation enhances the competitiveness of SMP products and distinguishes them with the products based on other technologies. By selecting a right commercially available engineering polymer, instead of using a newly developed special SMP, we can not only dramatically lower down the cost, but also produce anti-counterfeit shape memory labels with the required activation temperature.

#### 3.2.1. Tactual Sensation

As shown in the shape memory anti-counterfeit label in Figure 5, embossing and debossing are associated with out-of-plane programming of SMPs. If we use commercially available engineering polymers, such as polycarbonate (PC) and PMMA sheets, as the substrate, after embossing at room temperature using a Vickers indenter (for PC, refer to Reference [24]) or hot compressed using a special mold (for PMMA, refer to Reference [28]), concave or convex impression is produced. After heating to over their respective *T*_g_ (≈142 °C for PC [24] and ≈115 °C for PMMA [30]), their surfaces become flat. In addition to heating to flatten, heating to appear provides not only vision, but also tactile sensation, which differs from many currently used anti-counterfeit technologies. Through a process of indentation and polishing [1,64,90], an array of different shaped protrusions appears upon heating. The size of the protrusion can be as small as a few micrometers. In addition to heating, via indentation and then immersion in ethanol, micro protrusion (lens) of different sizes and shapes appear atop PMMA due to the stress enhanced swelling effect [91,92].

**Figure 13:**
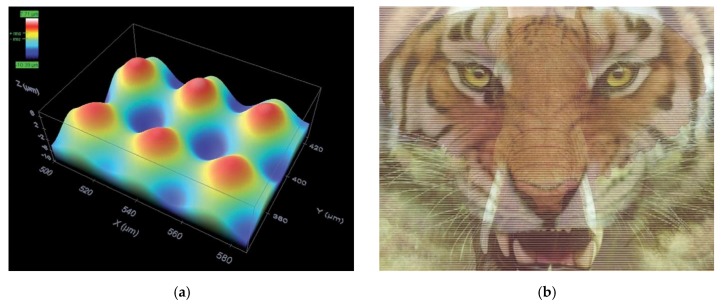
Array of protrusive micro lens which disappears upon heating (**a**) (reproduced from [8] with permission) and without micro lens array the image is a combination of tiger and elephant (**b**).

To increase the difficulty in replication, it might be designed in such a way that upon heating, the pattern of a special micro protrusion array on one side of a plastic film disappears, so that the underneath printed pattern on the other side loses the 3D effect. In Figure 13a, the micro lens array is produced atop a piece of pre-compressed optical polymer film using laser through a micro lens for heating of the required locations only. Such a micro lens array effect has been applied to realize 3D with naked eyes. The underneath image is specially designed to have more than one pictures integrated together (e.g., in Figure 13b). Through this micro lens array, from different viewing angles, we see different individual images (e.g., elephant or tiger in Figure 13b). If the surface becomes smooth upon heating, the image appears as a mixture of elephant and tiger. The difficulty to perfectly match the image on one side to the micro lens array on the other side is a big technical challenge, if anyone attempts to reproduce the 3D effect in a cost-effective way. Customized feature, which appears upon heating, may be produced by 3D printing as an additional security measure [79]. In Reference [15], it is reported to use SMP as a platform to integrate shape memory function and photo-imaging together for high-security information storage. 

**Figure 14:**
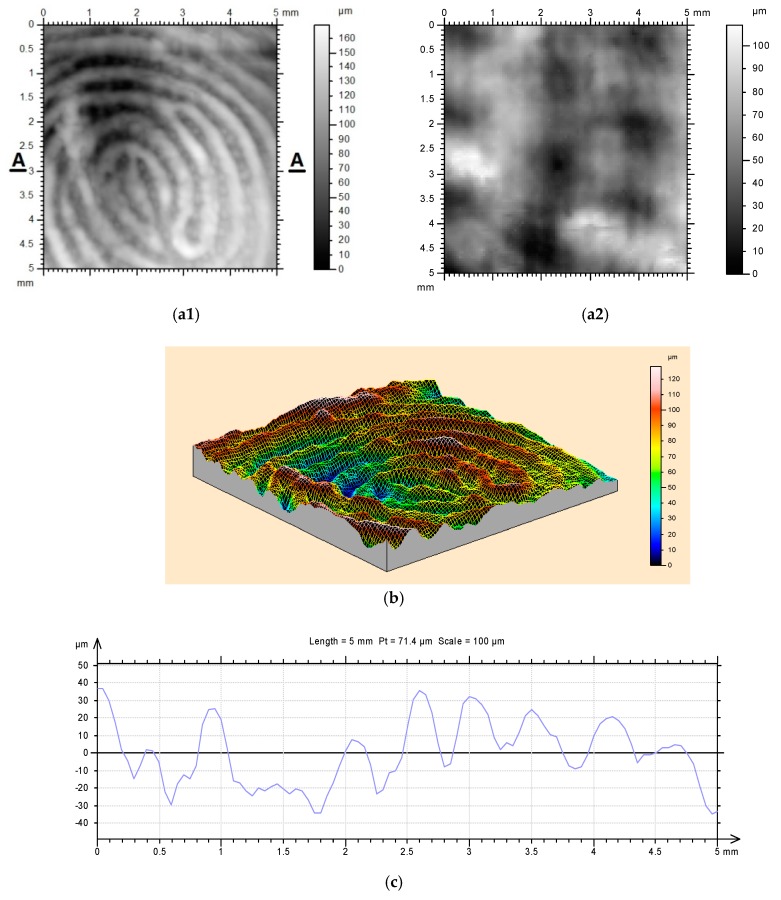
3D finger-print (2% cross-linked poly-ε-caprolactone (PCL). (**a**) Top view of 3D scanning result. (**a1**) After finger impression; (**a2**) after heating, the finger-print disappears. (**b**) 3D finger-print. (**c**) A-A section of finger-print.

As mentioned in Section 3, a highly interesting feature of PCL is that it is able to crystallize/harden at around body temperature after being heated to over its melting temperature, which is about 60 °C. Furthermore, at around body temperature to room temperature (about 20 °C), the crystallization process takes a few minutes to finish. This feature provides us with abundant time for programming to achieve comfort fitting in those applications, where direct contact with the human body is required [23], such as, commercial shape memory splints to fix fractured bone or to stop snoring (e.g., myTAP™ from Airway Management, USA). After cross-linking or blending with other polymers to result in SMHs (in which PCL works as small inclusions), we can achieve both high shape fixity ratio and high shape recovery ratio, and in the meantime maintain the above-mentioned special features of PCL that are particularly suitable for direct-body-contact-fitting. As demonstrated in Figure 14, the material can be heated to above its melting temperature and then our 3D finger print can be inscribed on the material at room temperature or around body temperature via a single gentle impressing of the finger on the surface of the material. The finger print can be fully removed by re-heating. The cross-section presented in Figure 14c reveals that the fluctuation in the depth of our finger print (adult) is about 70 μm, which is slightly less than that in bank coins and the commercial anti-counterfeit label as shown in Figure 5.

**Figure 15:**
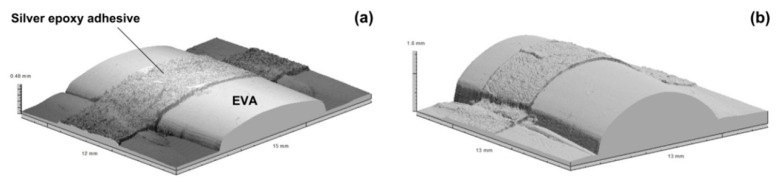
3D surface scanning results of ethyl-vinyl alcohol (EVA) after programming and coating with silver epoxy adhesive (**a**) and after heating for shape recovery (**b**). The height of the EVA protrusion increases from 0.48 mm to 1.6 mm after heating.

The phenomenon for the formation of protrusions upon heating can be utilized as a temperature sensor as well. Similar to that in Figure 11, in which an electrical circuit breaks if over-heating happens, in Figure 15, the height of a protrusion increases remarkably when it is heated to over a critical temperature, which might be tailored by programming, so that the silver epoxy adhesive atop of the protrusion fractures. Consequently, the electrical circuit, in which the silver epoxy adhesive is part of the circuit, shuts down. Since the glass transition in most polymers is sensitive to the equivalent time-temperature effect [93], melting transition could be a better choice in such applications. Furthermore, based on the concept of shape memory hybrid (SMH) [8,12], other non-polymeric materials, such as metals, could be used as the transition part to achieve a narrower transition temperature range and less sensitive to the equivalent time-temperature effect [1,94].

#### 3.2.2. Visual Sensation

In uniaxial stretching of many polymers at low temperatures, necking and propagation can be observed (e.g., PC in [24]). After this process of programming, PCL may change from opaque (for thick piece) or translucent (for thin piece) to transparent due to stress induced crystallization [29]. After subsequent heating, PCL becomes opaque or translucent again. This phenomenon (transition from opaque/translucent to transparent upon severe straining and upon subsequent heating from transparent back to opaque/translucent) may be utilized for anti-counterfeit applications, so that once heated, the feature behind these polymers becomes invisible (thick piece) [95]. While this transition phenomenon is limited to some specific polymers, the stress whitening effect (even silver streaks) is commonly observed in many polymers. As reported in References [59,96], folding a piece of write-on transparency film, which is PVC based, results in a white line, which can be removed by heating to above its *T*_g_ (about 60 °C). If examined under a microscope, we can see many micro lines within the white line and even the structural coloring effect can also be observed. Most of these lines disappear after heating, and so is the structural coloring effect. The diameter of the fibers produced via electro-spinning can reach sub-micron scale [97,98]. Even the bulky polymer itself is transparent, the very thin mat produced by electro-spinning could be fully opaque and even in shiny silver color. Upon heating, such a mat made of, for instance, above mentioned PCL [29] or TPU 265A [95], becomes almost transparent.

**Figure 16:**
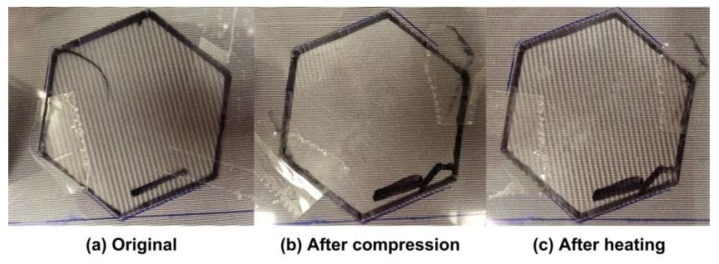
The shape memory effect (SME) in a 75-line per inch (LPI) PET lenticular lens sheet placed atop a piece of paper with printed regular line pattern. (**a**) Original lenticular lens sheet; (**b**) after compressing of lenticular lens sheet at high temperatures; (**c**) after heating of lenticular lens sheet for shape recovery.

PET is a popular engineering polymer used in a wide range of applications, such as substrate of RFID, printing transparency film, heat shrink film, and optical lens (such as lenticular lens and Fresnel lens), etc. Its *T*_g_ is typically around 70 °C. The heating-responsive SME of a commercial 75-line per inch (LPI) PET lenticular lens sheet, which is placed atop a piece of paper with printed regular line pattern, is demonstrated in Figure 16. In Figure 16a, we can see regular parallel lines atop the original sheet. After heating and compression to flatten the lens, the lines observed in Figure 16a largely disappear Figure 16b. Heating to over 80 °C again results in the return of the same parallel lines (Figure 16c) as in Figure 16a. The underlying mechanism behind this is the heating-responsive SME in the lenticular sheet and the Moiré effect.

**Figure 17:**
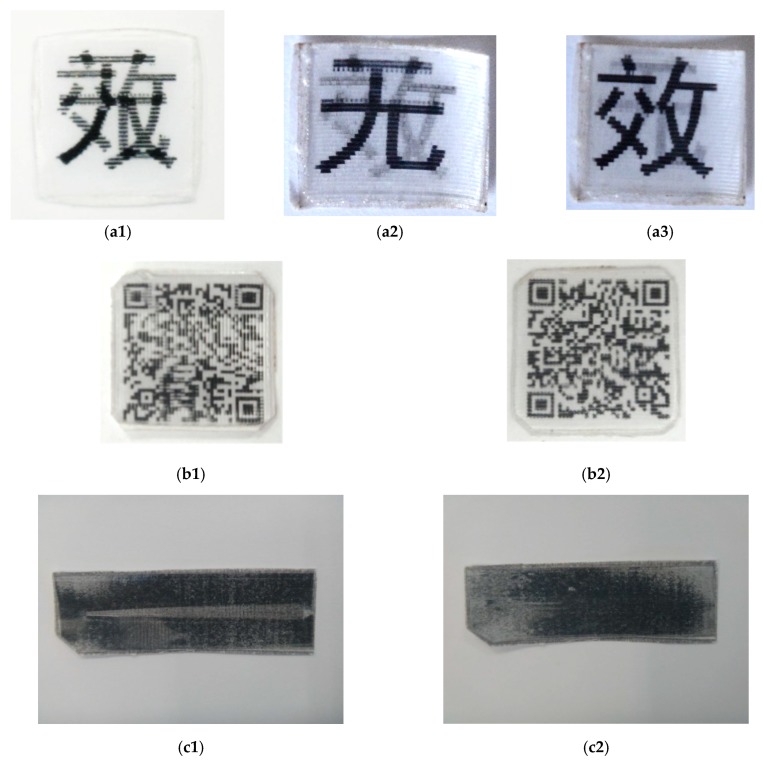
Three anti-counterfeit labels using 75-line per inch (LPI) PET lenticular lens sheet. (**a**) “Void” label. (**a1**) Before heating, the word is not easily recognizable; (**a2**) and (**a3**) after heating, viewing from right and left, respectively, we can see two Chinese words indicating void. (**b**) quick response (QR) code label. (**b1**) Before heating, not recognizable by smartphone; (**b2**) after heating, smartphone can read and show the meaning of the QR code. (**c**) Color change label. (**c1**) Before heating, the middle area appears as grey; (**c2**) after heating, the color is uniform on the whole surface.

Three anti-counterfeit labels developed using 75LPI PET lenticular lens are presented in Figure 17. Figure 17a is a void label, in which before heating, we cannot easily recognize its meaning (Figure 17(a1)), while after heating, viewing from right (Figure 17(a2)) and left (Figure 17(a3)) result in two Chinese characters, meaning void. Figure 17(b1) looks like an unreadable QR code, which after heating (Figure 17(b2)) and scanning using a smartphone, results in the appearance of the following sentence.

This label is void. There is a possibility that it has been heated over 80 degrees Celsius.

In Figure 17(c1), we can see a grey line in the middle of the black label, which disappears after heating (Figure 17(c2)). In a similar way, we can produce color change label, i.e., after heating, a seemingly uniformly colored label becomes having two different colors, depending on the angle of viewing. Such an approach may be extended to the currently used four-color-process for mass color printing to produce view angle dependent coloring effect and even 3D effect with naked eye.

**Figure 18:**
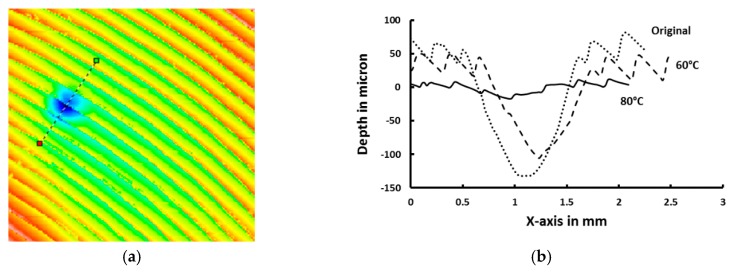
The shape memory effect (SME) in a commercial Fresnel lens. (**a**) Surface profile of Fresnel lens after indentation. Dashed line indicating the cross-section to be analyzed in (**b**); (**b**) cross-sections of Fresnel lens after indentation, and upon heating to 60 and 80 °C, respectively.

A Rockwell Hardness Tester DXT-3 is used to make an indent on a piece of Fresnel lens at room temperature. A spherical head is used for impression with a maximum load of 15 Kg. Figure 18a is 3D scanning result after indentation. In Figure 18b, the cross-sections along the dashed line indicated in Figure 18a are compared for three occasions, namely, after indentation, upon heating to 60 °C and then 80 °C. It can be concluded that unlike the above-mentioned lenticular lens, in which the shape of the lens is permanent, the profile of this Fresnel lens is most likely produced by hot embossing at around 80 °C. Thus, upon heating, not only the indent, but also the original feature of Fresnel lens, disappears.

As reported in Reference [8], the SME is a phenomenon even at nano-scale for many engineering polymers. Hence, micro-features can be produced atop SMPs via, for examples, nano-imprinting in a roll-to-roll manner [59,96] or self-wrinkling [62], so that we can see some interesting phenomena at this dimensional scale, such as the structural coloring effect [79].

#### 3.2.3. Multiple-Shape Memory Effect (SME)

As discussed in Section 2.2, the multiple-SME is meant for that between the programmed (temporary) shape and the permanent shape, there are one or more intermediate stable shapes, which can be precisely controlled, during shape recovery. As proved in Reference [19], the multiple-SME can be achieved in any SMP even based on only one transition, which could be the glass transition or melting/crystallization transition. Thus, at least one intermediate shape, i.e., the triple-SME, can be easily obtained within a normal transition temperature range of polymers (about 20–30 °C) by, for instance, step-by-step programming at different temperatures during cooling. From application point of view, the advantage of a narrow temperature range between the intermediate shape and the permanent one in the triple-SME is apparent in, for example, anti-counterfeit labels, as now we can use the intermediate shape as a kind of water-mark [30], which cannot be seen if over-heated.

**Figure 19:**
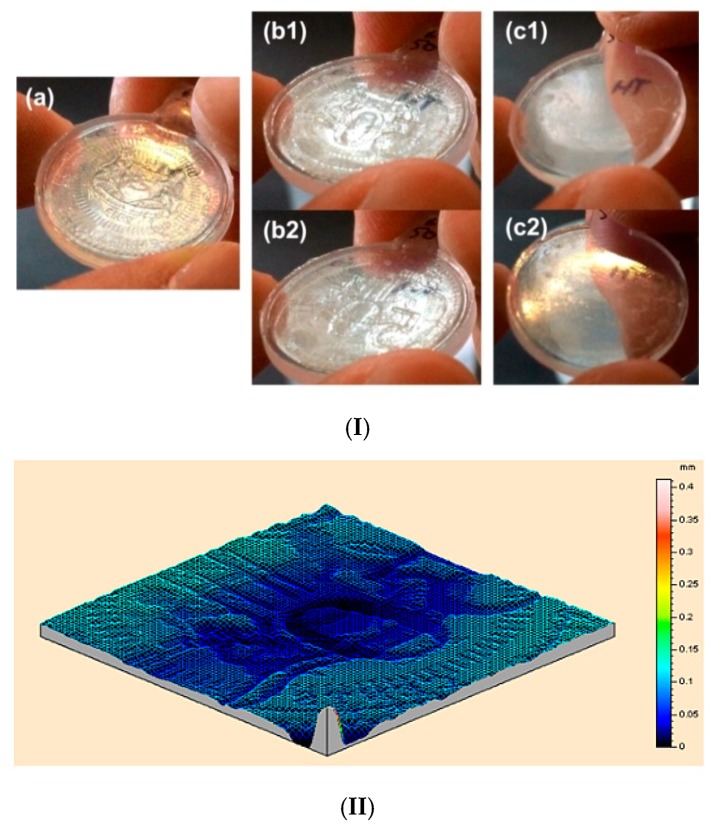
Anti-counterfeit label with a water mark. (**I**) (**a**) After programming; (**b1**,**b2**) after heating in 55 °C water (view from different angles); (**c1**,**c2**) after heating in 90 °C water (view from different angles). (**II**) 3D surface scanning result of the water-mark.

In Figure 19I, the same area of a piece of polymer is indented twice at different temperatures using the different sides of a coin in each step. In the subsequent heating process, the impression of one side of the coin (mirror image) switches to the other side of the coin (also mirror image) after 1st time of heating to 55 °C. Upon 2nd time of heating to 90 °C, the surface becomes flat. Figure 19II shows that the depth of the water-mark is less than 0.1 mm as expected. 

Flexibility is highly in demand in many types of non-rigid sensors, such as stick-type of labels, in order to avoid the problem of easy fracture, which is indeed a major problem in the SMP label reported in Figure 5. Rubber-like polymeric SMHs reported in Reference [85] are made of silicone mixed with a EVA based melting glue at high temperatures and then adding in agent for curing. A perfect combination of super-elasticity, which is tunable, and excellent heating-responsive SME enables labels made of such SMHs not only flexible for bending and twisting, but also stretchable for significant elongation. An improved version is recently reported in Reference [84], in which programming can be done at body temperature. Hence, it is applicable in direct contact with our skin during programming for comfort fitting.

**Figure 20:**
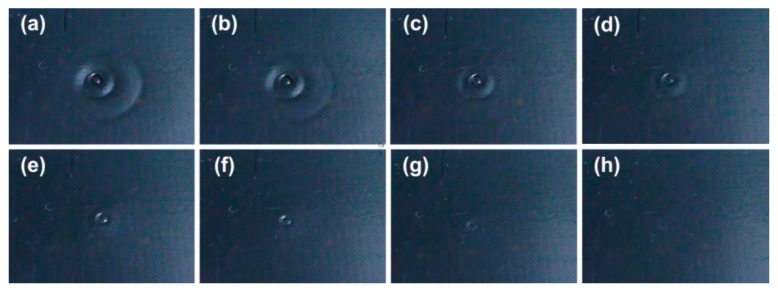
Shape memory polymers (SMP) temperature sensor to monitor actual over heating temperature. Upon gradual heating, three concentric circles disappear one by one from outside inward. (Reproduced from Reference [99]).

In order to monitor the actual over-heating temperature, which is very important in, for instance cold-chain logistics, in order to determine the actual quality of the good, in Figure 20, a piece of SMP temperature label is impressed at three different temperatures to result in three concentric circles (Figure 20a). Upon gradual heating, the circles disappear one-by-one from the outside toward the inside (Figure 20b–h). Thus, after calibration, based on the number of remaining circles, we can estimate the previous highest over-heating temperature. The underlying mechanism in this application is the TME, in which, same as that in Figure 19, the condition is that the maximum programming strain in each programming step should be small, so that the shape recovery in each step is mostly or even fully completed at around each corresponding programming temperature [19].

Step-by-step programming is rather tedious, particularly for more than one intermediate shape as demonstrated in Figure 20. While for those materials with multiple transitions, one-step programming for the multiple-SME is achievable [32], if the programming strain is larger, shape recovery cannot be finished or mostly finished upon heating to the previous programming temperature. Thus, we may utilize this phenomenon to develop the quasi-multiple-SME with only one step in programming based on one transition.

**Figure 21:**
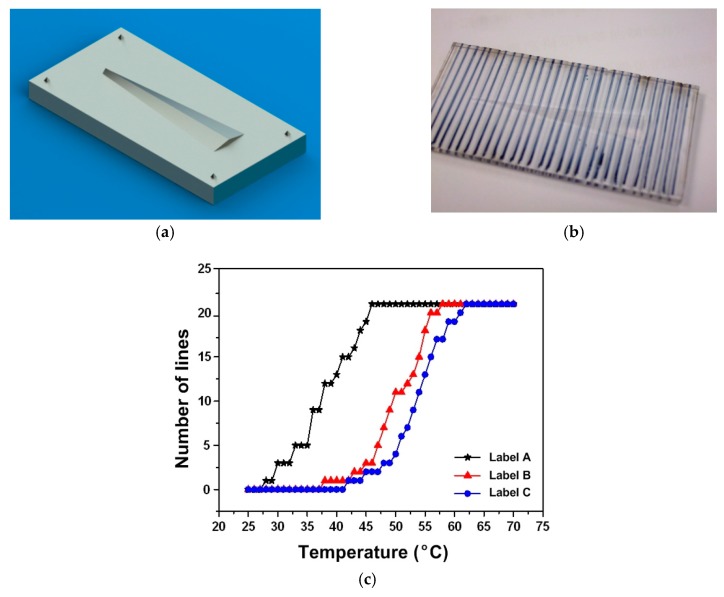
Designed mold (**a**), ethyl-vinyl alcohol (EVA) plate after impression (**b**), and (**c**) heating temperature vs. number of recovered parallel lines relationships in three labels prepared using different processing parameters (different programming temperature and different compressive force).

In Figure 21a,b, a mold with one single protrusion (variable triangle cross-section) atop is used to impress on a piece of EVA plate with pre-marked parallel lines atop. Upon gradual heating, shape recovery starts from one end and moves toward the other, and thus, more and more lines become straight again. As such, the shape recovery progress, which is related to the highest heating temperature, can be revealed directly by counting the number of recovered parallel lines within the indented area. Figure 21c presents typical experimental results of three such labels programmed using different processing parameters (different programming temperature and different compression force). It is obvious that by means of increasing the density of the parallel lines, better heating temperature estimation is expected. In Reference [79], a resolution of better than 0.5 °C between 80 to 82 °C is reported in a piece of polystyrene (PS) plate after impression using a specially designed lenticular mold. This result is better than many commercially available products, for instance, Thermax Level Strip Indicators and Tempilabel (from Tempil), which are only able to indicate a couple of pre-set temperatures.

#### 3.2.4. Hydrogel for Wetting Sensors

So far, all above mentioned applications are based on the heating-responsive SME. As water is easily accessible, various reversible water-activated anti-counterfeit labels have been in the market for some years. On the other hand, cost-effective wetting sensors, such as labels to indicate whether a particular item has been wetted by water and then dried back, are also useful.

According to Reference [12], the water-responsive SME should be an intrinsic feature of almost all dry hydrogels. In this section, instead of using some specially developed hydrogels, we use a commercial glue, namely Redox Glue 40, which is one of the glues that are commonly used as stationery in office, to demonstrate the feasibility that cheap commercial hydrogels can be utilized for wetting sensors. 

**Figure 22:**
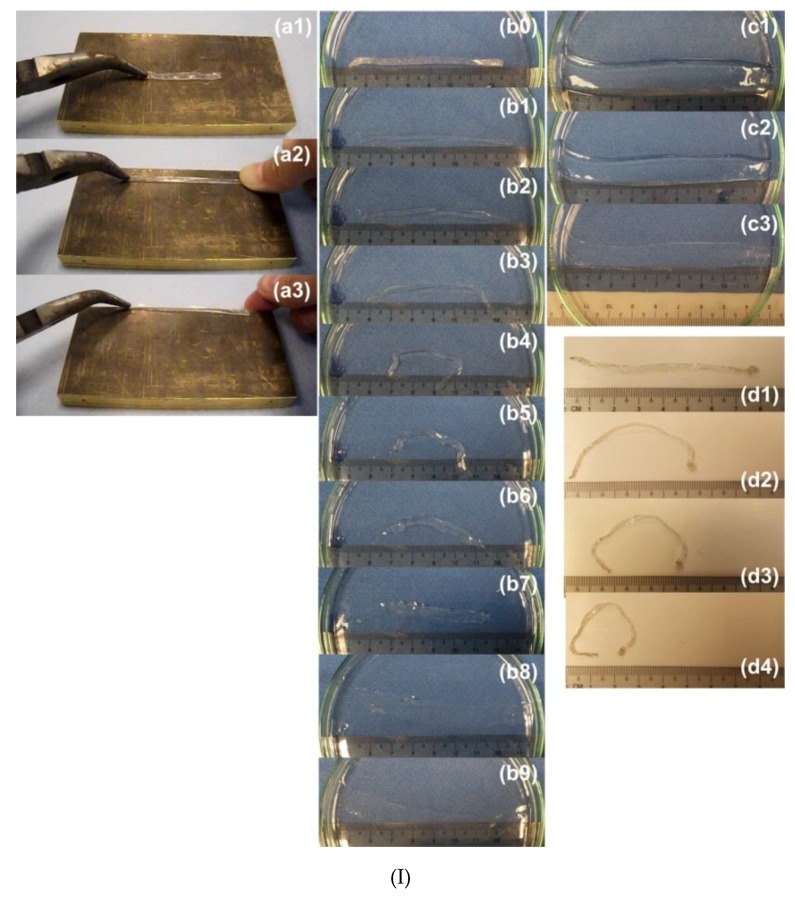

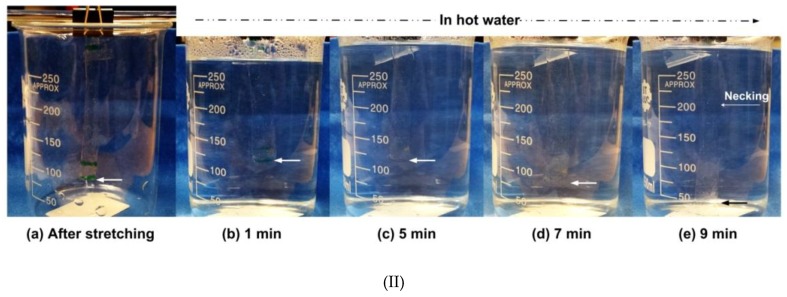
The shape memory effect (SME) in Redox Glue. (**I**) (**a**) Pre-stretching of strip (**a1**) after heating by hair dryer (**a2**) and then touching cold part of copper plate for quick cooling (**a3**); (**b**) (horizontally positioned) pre-stretched strip in room temperature water. Buckling due to water-induced shape recovery (**b3**–**b6**) and then swelling (**b6**–**b9**); (**c**) drying in air (shrinkage); (**d**) buckling upon further drying in air due to uneven shrinkage. (**II**) (Vertically positioned) pre-stretched strip in hot water. (**a**) After immersing in room temperature water; (**b**) shrinkage after 1 min due to water-responsive SME; (**c**) further shrinkage after 5 min; (**d**) stretching/swelling after 7 min in water; (**e**) necking due to stretching by self-weight. (White/black arrow indicates the position of its bottom end).

The SME of the dried glue, which is prepared by drying in air (relative humidity 50%, 25 °C), is demonstrated via two experiments. In the first experiment (Figure 22I(a)), a piece of dry strip is pre-heated and then stretched. After that, the pre-stretched strip is placed in room temperature water. A series of snapshots are presented in Figure 22I(b). Same as in Reference [54], buckling is observed, followed by swelling upon further wetting in water. Drying in air causes the strip to shrink (Figure 22I(c)). However, upon further drying, it becomes unstable again due to significant gradient shrinkage within its cross-section, which induces buckling (Figure 22I(d)). Different from that in Figure 22I(b), in which the sample is placed horizontally, in Figure 22II, a piece of pre-stretched strip is placed vertically into hot water. The bottom end of the strip is indicated by a white/black arrow. We can see that the strip quickly shrinks (due to the heating-induced SME) and then extends (due to swelling and self-weight stretching). After nine minutes, necking is observed (location is indicated), immediately followed by fracture at that point. 

**Figure 23:**
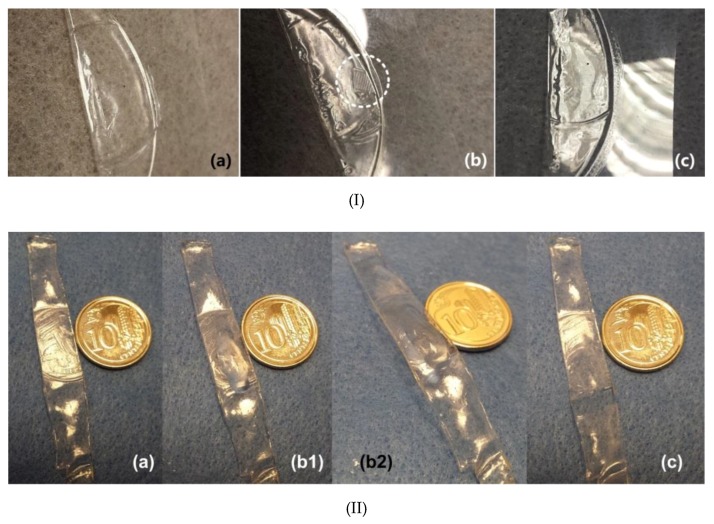
Dry Redox Glue based sensors. (**I**) Heating activated recovery. (**a**) Original; (**b**) after impression at high temperatures; (**c**) after heating, the impression disappears. (**II**) Water induced shape recovery. (**a**) After impression at high temperatures; (**b**,**c**) after slight wetting by water droplet; (**d**) after drying, the impression disappears.

Since the SME induced by room temperature water (water-responsive) and hot water (heating-responsive) is verified in Figure 22, this commercial glue is confirmed to be applicable for both heating sensor and wetting sensor applications. Two experiments are reported here for demonstration of such applications. A piece of dried glue strip (Figure 23I(a)) is impressed using a coin at high temperatures (Figure 23I(b)). The impression can be fully removed after heating (Figure 23I(c)). Another piece of dried strip is also impressed at high temperatures using a coin (Figure 23II(a)). A water droplet is used to cover the impressed area, which causes the strip to bend upwards due to swelling in the top surface (Figure 23II(b,c)). After drying, the strip returns back, and the coin pattern is also removed from its surface (Figure 23II(d)).

## 4. Summary and Outlook

**Figure 24:**
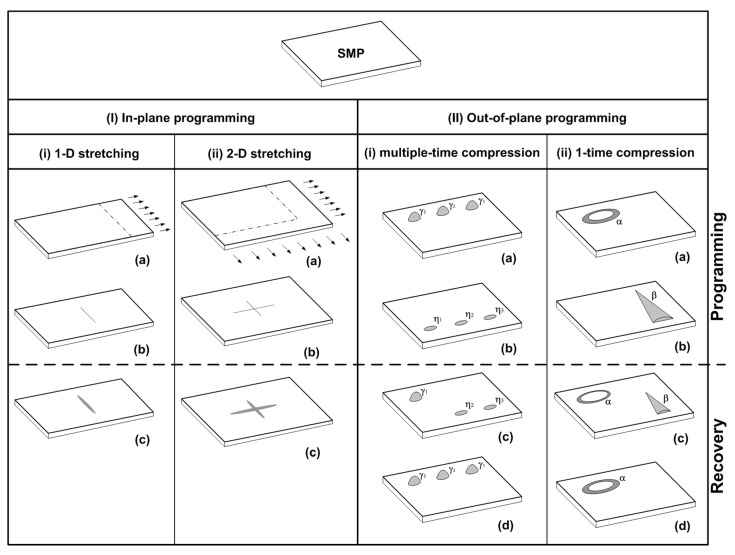
Summary of the main approaches to utilize the shape memory effect (SME) in polymers for heating-responsive sensors.

We have demonstrated a range of possible ways to utilize the SME in polymers for sensor applications. A summary of the main approaches based on the heating-responsive SME is presented in Figure 24. For relatively thinner polymeric substrates (e.g., less than 100 μm), after in-plane programming via 1D or 2D stretching (I-i-a and I-ii-a), a sharp blade can be used to cut the substrates (I-i-b and I-ii-b). Upon heating the cut becomes wider and easily detectable (I-i-c and I-ii-c). Many commercial polymeric substrates (with 1D/2D pre-stretching) are readily available to be used for such applications. For relatively thicker substrates (e.g., over 100 μm), out-of-plane programming may also be applied. The main advantage of out-of-plane programming is that we can achieve the multi-SME for step-by-step recovery in a controllable manner via either multiple-time programming or one-time programming. In both (II-i-a) and (II-ii-a), the substrates are produced with multiple (γ_1_, γ_2_ and γ_3_) or single (α) protrusion. All these protrusions are permanent and carefully designed with special geometric dimensions. Programming via compression is done either in multiple steps at different temperatures to gradually remove these protrusions (γ_i_, I = 1,2,3) and simultaneously, one-by-one to produce multiple indents (η_i_, I = 1,2,3) (II-i-b), or in one step at one temperature to remove the protrusion (α) and in the meantime, to introduce a new indent (β) (II-ii-b). In the subsequent recovery process, upon gradual heating, protrusions (γ_i_) reappear one-by-one, while indents (η_i_) disappear one-by-one; or protrusion (α) gradually appears, while indent (β) gradually disappears. Similar approaches can be extended to water or moisture-responsive applications using water/moisture-responsive SMPs. 

In Table 1, typical SMPs available in the commercial market are summarized. Their main features and possible suppliers are included. References are also provided to reveal the details of their shape memory performance for interested readers. This table is by no means an exhaustive list, as many commercial polymers mentioned above (e.g., PET, PE, PVC, redox glue, etc.) are not included. We should take note that the actual shape memory performance of a particular type of commercial polymer varies according to the supplier, since the manufacturers have mostly done some modifications in order to improve the performance for the targeted applications.

**Figure 25:**
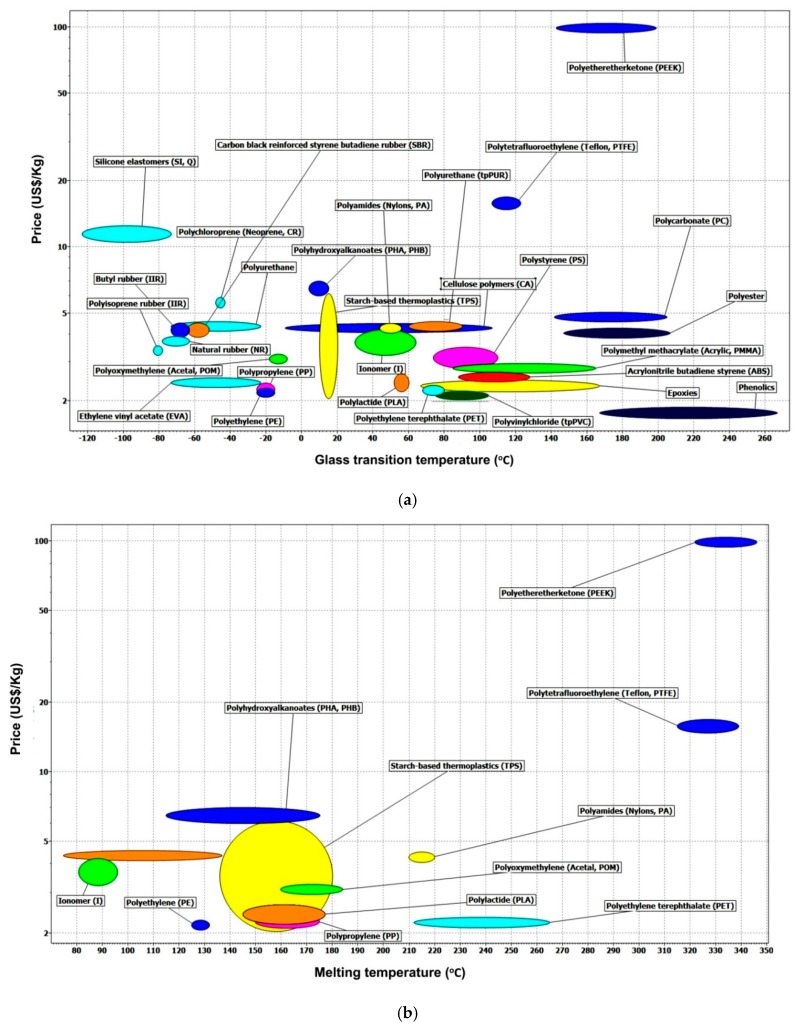
*T*_g_ vs. price chart (**a**) and *T*_m_ vs. price chart (**b**) of typical engineering polymers (produced by Cambridge Engineering Selector, http://www.grantadesign.com/). Logarithm scale is used for price.

As mentioned above, price is a very sensitive topic in many engineering applications. While the actual processing cost depends on many issues, in Figure 25, the charts of *T*_g_ and *T*_m_ vs. price (in US$/Kg, logarithm scale) of some typical engineering polymers are presented. These two charts are produced by Cambridge Engineering Selector with the most recently updated data. Based on these two charts, we can select the right polymers for the required working conditions (e.g., activation/transition temperature) and the actual processing approach (e.g., how to fix the permanent shape/pattern). Note that many amorphous polymers, such as, PC, PMMA, and ABS, have no melting temperature, but have excellent heating-responsive SME. PEEK, and PTFE are far more expensive than many other engineering polymers, while PET, PP, PE, and PLA are among the cheapest. 

Although we have seen good progress in recent years in R&D on SMPs, apart from continuous development in new SMPs with various interesting features, from the aspect of engineering applications, SMP actuators are more emphasized by most of the researchers. Anti-counterfeit label is seemingly one of the least successful SMP sensor products in the market so far. Since the cost-effectiveness is the most important requirement for any commercial products, due to relatively high material cost, SMP anti-counter label appears to be not so competitive in the current market when competing with other anti-counterfeit products based on other technologies. If we use mass produced engineering polymers, for instance, the polymers used in conventional labels/packaging and heat-shrink applications, to replace these special SMPs, the cost of such sensors is expected to be lowered significantly. According to Figure 25, most of the recyclable plastics used in our daily life (PET, high density polyethylene (HDPE), PVC, low density polyethylene (LDPE), PP, PS, and ABS, etc.) can be utilized as the raw material in an environmentally friendly and cost-effective way.

Since the SME may be considered as a generic feature of many engineering polymers, as demonstrated experimentally by the many examples above, the products made of these polymers (such as hologram sticker, lenticular lens, and heat shrink film) can be easily enabled with the sensing function via shape/surface pattern switching in response to, for instance, heat, with only a small amount of extra cost. This could be the direction for the next step of our R&D. On the other hand, many polymer-based new technologies, e.g., roll-to-roll nano-imprinting, electro-spinning, and 3D printing, have been well developed in recent years. These technologies are able to enhance the functions of the SMPs in many sensor applications.

The excellent SME observed in a great number of polymers indeed provides many opportunities for them to be considered as potential candidates in not only actuation applications, but also sensing applications. However, in order to compete with other exciting or new technologies, we should pay more attention to the cost-effectiveness of the SMP products in order to enhance the chance of their success in the commercial market. 

Depending on the actual application and the working conditions, the equivalent time–temperature effect may be considered or utilized in the selection of polymers for sensor applications. The influence of the equivalent time-temperature effect may be less remarkable in melting transition-based SME for some polymers. SMH may be an alternative to get rid of or minimize this effect.

SMPs with a reversible shape switching feature via either mechanical two-way SME (in which the elastic component to reset the temporary shape is outside of the SMP) or material two-way SME (in which the elastic component to reset the temporary shape is within the SMP) may provide another solution to many sensor applications with the requirement of automatic re-programming for multiple shape memory cycles. However, their long-term repeatability, e.g., over 1000 s of thermal cycles or more, needs to be carefully examined.

## 5. Conclusions

As discussed in this brief review, the SME, which is different from the SCE, has many potential applications. There are some different possible underlying mechanisms for the SME in polymers. On the other hand, different types of shape memory performance can be achieved via programming in a tailored manner. 

Accordingly, we show that many commercially available engineering polymers have excellent SME, and furthermore, a lot of existing polymeric products can be easily modified to have the ability for shape/surface pattern switching upon heating or wetting by, for instance, water. Hence, these materials/products can be programmed either through in-plane stretching or out-of-plane impression in one step or multiple steps to have the additional function for visual sensation and/or tactual sensation, which is the most interesting feature of SMPs and is different from many existing sensors based on other technologies. 

Multiple experiments, in particular for anti-counterfeit and over-heating/wetting monitoring, are presented here to demonstrate the feasibility of integrating the SME in polymers into a wide range of existing technologies and commercial products (e.g., holography, 3D printing, nano-imprinting, electro-spinning, lenticular lens, Fresnel lens, QR/bar code, Moiré pattern, FRID, structural coloring, etc.) for cost-effective sensors in real engineering practice.

## Figures and Tables

**Table 1:** Summary of typical commercial shape memory polymers (SMPs).

Polymer (Reference)	Activation Temperature	Main Features	Possible Supplier
ABS [62]	*T*_g_: 105 °C	Thermo-plastic Excellent heating-responsive SME	Professional Plastics Pte, Ltd., Singapore
EVA [26]	*T*_g_: 60 °C	Thermo-plastic; Excellent heating/chloroform-responsive SME	Polyfluo Asia Private Limited, Singapore
PC [24]	*T*_g_: 142 °C	Thermo-plastic Excellent heating-responsive SME	Polyfluo Asia Private Limited, Singapore
PCL [29]	*T*_m_: 55 °C	Thermo-plastic; Bio-degradable Excellent heating-responsive SME Programmed at low temperatures	Perstorp, UK
PEEK [25]	*T*_g_: 155 °C	Thermo-plastic Excellent heating-responsive SME	Evonik Degussa through Professional Plastics Pte, Ltd. (Singapore)
PLA [47]	*T*_g_: 65 °C	Thermo-plastic; Bio-degradable Good heating-responsive SME	eSUN, PR China (filament for 3D printing)
PMMA [28,30,91]	*T*_g_: 115 °C	Thermo-plastic; Good heating/ethanol-responsive SME	Ying Kwang Acrylic, Singapore
PS [12,65]	*T*_g_: 65 °C	Thermo-set; Good heating/acetone-responsive SME	Cornerstone Research Group, Inc., USA
PTFE [27]	*T*_g_: 65 °C *T*_m_: 325 °C	Thermo-plastic Excellent heating-responsive SME Two-step recovery upon heating	Polyfluo Asia Private Limited, Singapore
PU [52]	*T*_g_: 35~65 °C	Thermo-plastic/thermo-set Biocompatible; Excellent heating/ethanol/water-responsive SME	SMP Technologies, Inc., Japan
PVA [47]	*T*_g_: 30 °C	Thermo-plastic Excellent heating-responsive SME Water-responsive SME	eSUN, PR China (filament for 3D printing)
TPU [95]	*T*_m_: 55 °C	Thermo-plastic/vitrimer Excellent heating-responsive SME	Taiwan PU Corporation (TPUCO), Taiwan
